# Mechanical Ventilation and Survival Without Major Morbidity in Very‐Low‐Birth‐Weight Infants: A Multicenter Study

**DOI:** 10.1155/ijpe/4067076

**Published:** 2026-10-14

**Authors:** Alberto Toso, Daniela Kuzmanic, Angelica Dominguez, Alvaro Gonzalez, Cecilia Herbozo, Pablo H. Brener Dik, Luis A. Ahumada, Alvaro Mendez, Claudia Aburto, Jose L. Tapia, NEOCOSUR Neonatal Network

**Affiliations:** ^1^ Department of Neonatology School of Medicine Pontificia Universidad Catolica de Chile Santiago Chile uc.cl; ^2^ Red de Salud UC CHRISTUS Santiago Chile; ^3^ School of Public Health Faculty of Medicine Pontificia Universidad Católica de Chile Santiago Chile uc.cl; ^4^ Universidad Peruana Cayetano Heredia Hospital Cayetano Heredia Lima Peru upch.edu.pe; ^5^ Department of Pediatrics Division of Neonatology Hospital Italiano de Buenos Aires Buenos Aires Argentina hospitalitaliano.org.ar; ^6^ Maternidad Provincial Brigadier Gral. Juan Bautista Bustos Cordoba Argentina; ^7^ Hospital regional de Talca Dr. Cesar Garavagno Burotto Talca Chile; ^8^ Universidad Catolica del Maule Talca Chile ucm.cl; ^9^ Hospital Dr. Guillermo Grant Benavente Concepcion Chile

**Keywords:** mechanical ventilation, morbidity, mortality, noninvasive ventilation, premature infants, very low birthweight infants

## Abstract

**Background:**

Mechanical ventilation (MV) is a frequent lifesaving therapy in very low birthweight (VLBW) infants, although it poses risks and may present complications.

**Objective:**

To evaluate the association between the use and duration of MV and survival without major morbidity (SWMM) in VLBW infants according to gestational age (GA).

**Methods:**

Multicenter retrospective study of prospectively collected data of premature infants weighing 500–1500 g and 24–30 weeks of GA, born between 2010 and 2019 at NEOCOSUR Neonatal Network centers. Baseline characteristics and neonatal outcomes were compared between ventilated and nonventilated VLBW infants. Stratification for each week of GA was performed. Adjusted odds ratios and 95% confidence intervals (CIs) were calculated for main outcomes by logistic regression, adjusted for birthweight, GA, sex, mode of delivery, respiratory distress syndrome (RDS), antenatal corticosteroids, small for gestational age (SGA), and Apgar scores. Infants with congenital anomalies and those who died before discharge were excluded.

**Results:**

A total of 7040 infants were included in the analysis. Among these, 4993 (70.9%) were ventilated and 2047 (29.1%) were not. Nonventilated infants were larger, healthier at birth, and presented fewer major adverse outcomes (*p* < 0.001). SWMM in ventilated patients was 35.9%, compared with 73.4% in nonventilated infants (OR = 0.35; 95% CI: [0.31–0.40]). SWMM of ventilated infants remained lower at all GA intervals after risk adjustment. MV duration was also associated with a decrease in SWMM; each additional day of MV was associated with 7.9% lower odds of SWMM (OR = 0.921; 95% CI: [0.913–0.930]).

**Conclusions:**

The use and duration of MV were associated with a significant decrease in SWMM in this population of VLBW infants, despite risk adjustment.

## 1. Introduction

Mechanical ventilation (MV) is a common and lifesaving therapy among very low birthweight (VLBW) infants. However, its use is associated with an increased risk of major comorbidities.

Bronchopulmonary dysplasia (BPD), the most common chronic morbidity of surviving very preterm infants, has been associated with MV since its original description [1]. The incidence of BPD has not changed much throughout the years, possibly because of the increased survival of smaller infants [2].

Although MV has been consistently associated with adverse neonatal outcomes, an important methodological challenge is that infants requiring MV are usually more critically ill, which may confound the observed associations. Therefore, distinguishing whether MV itself contributes to worse outcomes or whether it is simply a marker of illness severity remains complex.

The current best evidence portrays that a longer cumulative duration of MV accounts for the increased risk of chronic respiratory morbidity seen in these patients, especially BPD [3]. For instance, Brener Dik et al. [4] showed that each day of MV increased the association with moderate/severe BPD by 8%, regardless of other outcome measures included in the model. Additionally, Choi et al. [5] described that a longer cumulative duration of MV was associated with BPD, periventricular leukomalacia (PVL), retinopathy of prematurity (ROP) requiring laser photocoagulation, and increased length of hospital stay. Other studies have reported an association between the duration of MV and severe intraventricular hemorrhage (IVH) (Grades III–IV), necrotizing enterocolitis (NEC), and late‐onset sepsis [6–9].

The aim of this study was to describe the association between the use and duration of MV and survival without major morbidity (SWMM) among VLBW infants in a large multicenter setting, stratified by gestational age(GA), ranging from 24 to 30 weeks.

We hypothesized that the use and duration of MV will decrease the SWMM at all GAs.

## 2. Subjects and Methods

### 2.1. Study Design

This is a multicenter, observational, cohort study.

### 2.2. Data Collection

Data was obtained from the NEOCOSUR Neonatal Network database, which includes 32 centers from six South American countries (Argentina, Brazil, Chile, Paraguay, Peru, and Uruguay). The NEOCOSUR network provides a continuous database, registering information from all inborn VLBW infants in the participating centers. Centers and patient identities are kept anonymous. Biodemographic characteristics and outcome data of all VLBW infants born in these centers are prospectively registered, using predefined diagnostic criteria and online data entry.

### 2.3. Study Population

Data from all inborn infants with birthweight between 500 and 1500 g and GA between 24 and 30 weeks, born between 2010 and 2019, were included for the purpose of this study. MV refers to invasive ventilation through an endotracheal tube.

Infants who died prior to hospital discharge and those born with congenital anomalies were excluded.

### 2.4. Primary Outcome

SWMM was defined as survival to hospital discharge without any of the following major morbidities: BPD, severe IVH (Grades III‐IV), cystic PVL, NEC requiring surgery, ROP (Stages 3–5) requiring treatment, or late‐onset sepsis (LOS).

Severe IVH was diagnosed by cerebral ultrasound and included Grades III–IV according to Papile criteria; NEC confirmed by abdominal radiography (pneumatosis and/or perforation) or surgical findings; LOS, confirmed by positive cultures for bacteria, virus or fungi after 72 h of life; PVL diagnosed by white matter intraparenchymatous necrotic lesions seen by head ultrasound; ROP was diagnosed through eye examination by an ophthalmologist after 28 days of birth and classified in Stages 1–5 according to the International Classification and only included infants alive at 28 days; BPD, defined as oxygen use at 36 weeks′ postmenstrual age (PMA) or discharge, whichever came first [10].

Because this is an observational study, the indication for MV was not randomized and may reflect underlying illness severity. Therefore, MV should be interpreted as an exposure associated with disease severity rather than a causal factor.

This study was approved by our institution′s ethics committee (ID 220708001).

### 2.5. Statistical Analysis

Baseline characteristics and neonatal outcomes were compared among ventilated and nonventilated VLBW infants. Magnitude of effect for MV over main neonatal outcomes was obtained using logistic regressions. A multivariate model was built including all those factors with potential influence on the results based on the literature review and the group′s previous experience. Accordingly, antenatal corticosteroids, mode of delivery, birthweight, GA, small for gestational age (SGA), 1‐min Apgar score, 5‐min Apgar score, sex, and respiratory distress syndrome (RDS) were considered for adjustment. SWMM is shown for each week of GA, and for the duration of MV.

Incidence of SWMM by ventilation status was calculated for each week of GA.

Adjusted odds ratio and 95% confidence intervals (CIs) were obtained by logistic regression, adjusting for antenatal corticosteroids, mode of delivery, birthweight, GA, SGA, 1‐min Apgar score ≤ 3, 5‐min Apgar score ≤ 3, sex, and RDS.

In those who received MV, association of duration of MV and SWMM was calculated.

Significance level was set at 0.05. Statistical analyses were performed using SPSS 17.0 (Chicago, Illinois, United States).

## 3. Results

A total of 7828 preterm infants met the inclusion criteria. Of these, 788 infants had relevant missing information. Thus, 7040 infants were included in the analysis, with a mean GA of 28.0 ± 1.6 weeks and mean birthweight of 1078 ± 238 g. A total of 4993 (70.9%) were ventilated, and 2047 (29.1%) were nonventilated.

General characteristics, risk factors and main outcomes of the MV, non‐MV, and overall population, are shown in Table 1. Nonventilated infants had a higher proportion of antenatal corticosteroids use and female sex; they were also larger, had higher Apgar scores, and a significantly better SWMM (*p* < 0.001) (Table 1).

**Table 1:** Baseline characteristics and outcomes between ventilated and nonventilated patients. NEOCOSUR, 2010–2019.

	**MV**	**Non-MV**				
	**(** **n** = 4993**)**	**(** **n** = 2047**)**	**(** **n** = 7040**)**	**p**		

Baseline characteristics						
Antenatal corticosteroids use; %	86.0	91.3	87.5	< 0.001		
Mode of delivery, c‐section; %	75.8	74.8	75.5	0.382		
Birthweight (g); mean ± SD	1033 ± 236	1186 ± 205	1078 ± 238	< 0.001		
Gestational age; mean ± SD	27.6 ± 1.7	28.9 ± 1.1	28.0 ± 1.6	< 0.001		
SGA (Fenton); %	6.0	4.9	5.7	0.064		
1‐min Apgar score ≤ 3; %	22.2	3.4	16.7	< 0.001		
5‐min Apgar score ≤ 3; %	3.5	0.1	2.5	< 0.001		
Female; %	45.7	52.1	47.5	< 0.001		
RDS; %	96.1	73.0	89.4	< 0.001		

**Outcomes**				**p**	**Adjusted OR** ^**a**^	**95% CI**

SWMM; %	35.9	73.4	46.8	< 0.001	0.35	(0.31–0.40)
Severe IVH; %	10.4	1.5	7.8	< 0.001	4.37	(2.95–6.48)
NEC; %	13.4	4.2	10.7	< 0.001	2.57	(1.99–3.33)
Late onset sepsis; %	30.9	11.7	25.3	< 0.001	2.48	(2.09–2.94)
PVL; %	10.4	5.7	9.0	< 0.001	1.16	(0.91–1.47)
Oxygen at 36 weeks; %	37.3	8.2	28.8	< 0.001	3.21	(2.67–3.86)
Severe ROP; %	7.3	1.0	5.4	< 0.001	2.29	(1.38–3.79)

*Note:* Correction added on 20 October 2026, after first online publication: The alignment of the baseline characteristic “RDS; %” has been adjusted to appear correctly in this version.

Abbreviations: CI, confidence interval; IVH, intraventricular hemorrhage; MV, mechanical ventilation; NEC, necrotizing enterocolitis; OR, odds ratio; PVL, periventricular leukomalacia; RDS, respiratory distress syndrome; ROP, retinopathy of prematurity; SD, standard deviation; SGA, small for gestational age; SWMM, survival without major morbidity.

^a^Adjusted for antenatal corticosteroids, mode of delivery, birthweight, GA, SGA, 1‐min Apgar, 5‐min Apgar, gender, and RDS.

Use of MV ranged from 100% at 24 weeks to 51.5% at 30 weeks GA. Median use of MV in survivors was 6 days and ranged between 37 days at 24 weeks to 2 days at 30 weeks GA. SWMM was significantly higher in nonventilated patients and infants with an older GA (Figure 1).

**Figure 1:**
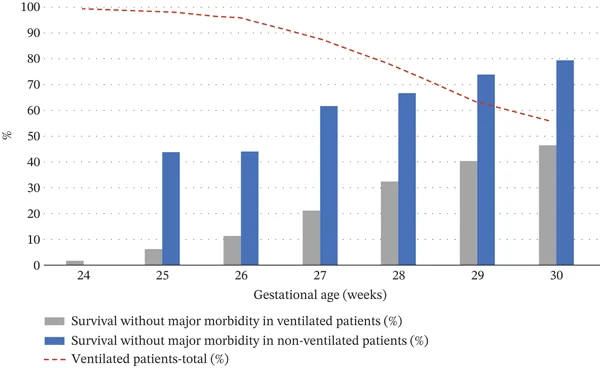
Survival without major morbidity in ventilated and nonventilated patients and total use of mechanical ventilation according to gestational age. NEOCOSUR, 2010–2019. Legend: The distribution of SWMM varied between weeks of gestational age in ventilated and nonventilated patients (both *p* values < 0.001). The distribution of MV varied between weeks of gestational age (*p* value< 0.001). SWMM, survival without major morbidity; MV, mechanical ventilation.

In addition, SWMM progressively decreased as the duration of MV increased across different strata: no MV, 1–3 days, 4–7 days, and > 7 days (*p* < 0.001) (Figure 2).

**Figure 2:**
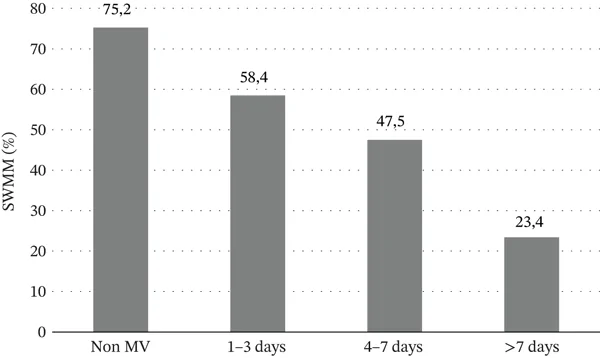
Survival without major morbidity stratified by duration of mechanical ventilation. NEOCOSUR, 2010–2019. Percentage of survival without major morbidity decreased along mechanical ventilation duration strata (*p* value for trend < 0.001). SWMM, survival without major morbidity; MV, mechanical ventilation.

Overall SWMM was lower among ventilated infants (OR = 0.35; 95%CI: [0.31–0.40]) after adjustment for perinatal variables. When stratifying by GA, SWMM among ventilated infants remained significantly lower than that for nonventilated infants, especially at younger GA (Figure 3).

**Figure 3:**
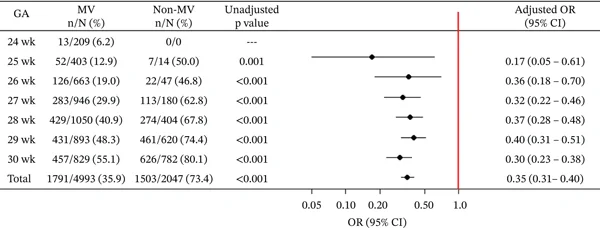
Survival without major morbidity at different gestational ages (weeks) with and without mechanical ventilation (MV). OR was adjusted for antenatal corticosteroids, mode of delivery, birthweight, GA, small for gestational age (SGA), 1‐min Apgar, 5‐min Apgar, sex, and respiratory distress syndrome (RDS). NEOCOSUR, 2010–2019. CI, confidence interval; GA, gestational age; MV, mechanical ventilation; OR, odds ratio; wk, weeks.

Among ventilated infants, MV duration was also associated with a significant decrease of SWMM, showing that each additional MV day was associated with 7.9% lower odds of SWMM (OR = 0.921; 95% CI: [0.913–0.930]) adjusted for perinatal variables.

## 4. Discussion

The main finding in this multicenter study is that the use and duration of MV were associated with lower SWMM at all GAs between 24 and 30 weeks.

Although infants had dissimilar baseline characteristics, the use of MV was associated with an increased incidence of major morbidities even after adjusting for main perinatal variables.

SWMM is increasingly used as a relevant outcome measure for VLBW infants in several neonatal networks [11–13]. The rationale behind this is that these morbidities are associated with an increased risk of long‐term severe neurodevelopmental impairment (NDI) [14]. In addition, these morbidities are predictors of long‐term adverse outcomes including cerebral palsy, cognitive delay, severe hearing loss, and bilateral blindness [15].

Our results are in line with recent studies that describe an association with impaired brainstem development, abnormal white matter maturation, and lower motor scores at preschool age; each additional day of MV predicts lower motor scores [16]. Moreover, every additional day of invasive MV increases the risk of NDI at 24 months [17]. Further research is required for a better understanding of the pathological mechanisms involved for this association [17]; however, it could be speculated that it may be due to the increased risk of major morbidities.

Other recent studies also show an association of a longer exposure of MV with increased risk of gastrostomy in preterm patients < 1500 g with BPD [18]. A study from Brazil finds that a longer duration of MV exposure is associated with the development of moderate to severe BPD, representing a worse outcome in infants ≤ 28 weeks GA [19].

Limiting invasive MV should be an important issue in the care of VLBW infants. Strategies such as minimally invasive techniques for surfactant administration and the use of noninvasive respiratory support have been shown to decrease the need for MV [20]. The INSURE strategy (intubation, surfactant administration, and extubation to continuous positive airway pressure(CPAP)) and more recently, less invasive surfactant administration through a thin catheter (LISA) both decrease the need for MV [21, 22]. A recent Cochrane review reports that LISA may also decrease the risk of BPD [22].

The use of noninvasive respiratory support, mainly by early CPAP, in systematic reviews and meta‐analysis has been shown to reduce (although modestly) the combined outcome BPD/death, as well as the need for surfactant and MV [23].

Other noninvasive initial respiratory support strategies for VLBW infants such as nasal intermittent positive pressure ventilation, nasal high frequency ventilation, or high flow nasal cannula still require more well‐designed trials regarding their effectiveness and safety.

A recent Chinese study compared two periods of time (2007–2011 and 2012–2016) in a population of VLBW infants (≤ 34 weeks GA). An improvement in perinatal practices was associated with an overall reduction of in‐hospital mortality. The use and length of exposure to MV decreased while the use and duration of CPAP increased. Associated with these, there was a significant increase in the proportion of survivors without major neonatal morbidity [24].

A quality improvement (QI) respiratory bundle has proven to be effective in decreasing the use of MV in very preterm infants from a single center study [25].

In addition, a restricted use of MV in preterm infants has proven feasible and might be associated with a reduced risk of the combined outcome death or NDI at 24 months corrected age [26].

Some studies favor extubation attempts in VLBW infants receiving MV on low ventilator settings, even when success is not guaranteed [3, 27].

Several interventions have proven useful for a successful extubation of preterm infants. These include routine caffeine administration, extubation to CPAP or nasal noninvasive ventilation [28]. The implementation of a weaning protocol may also decrease the duration of MV [29]. The use of late postnatal steroids has been shown to facilitate extubation; however, there is a lack of consensus regarding its benefits and risks [30].

This study has several limitations, besides being a retrospective analysis, although data are prospectively collected. First, severity of RDS, specific respiratory and ventilator parameters are not registered in our database. Similarly, the type of ventilator used and the ventilation mode (both invasive and noninvasive) are not registered.

Another limitation is that ventilated (MV) and nonventilated (non‐MV) infants differed in baseline clinical characteristics and risk profiles. Although we attempted to attenuate these differences through multivariate adjustment, residual confounding and confounding by indication cannot be excluded. Some components of SWMM, such as NEC requiring surgery, LOS, and moderate/severe BPD, may themselves increase the likelihood of requiring MV. Therefore, MV should be interpreted not only as an exposure associated with lower SWMM, but also potentially as a marker of greater illness severity.

Among the strengths of this study, it is worth emphasizing that our results are broadly generalizable as it includes a large number of infants from a multicenter middle‐income region setting, with very heterogeneous populations coming from six different countries. In addition, we analyzed our data stratifying by GA, which is very important given that the adverse outcomes related to prematurity vary by GA.

Our results suggest that every effort should be made to limit and shorten the duration of MV whenever possible. Clear intubation and extubation protocols should be followed when caring for VLBW infants. Clinicians should identify candidates for noninvasive ventilation strategies. Additionally, if MV is required, continuously re‐evaluate its use, emphasizing that each additional MV day counts in decreasing survival free of major morbidity.

Nevertheless, we cannot conclude from this study that noninvasive respiratory support would have been a better alternative for these infants, considering that MV may be a lifesaving therapy for some of them. However, an additional comment can be made regarding our data showing what seems a very high (70.9%) use of MV in these 24–30 weeks VLBW infants. In contrast, reports from the QI bundle study show a decrease from 46% to 27.6% in a cohort of infants < 32 weeks′ gestation [25].

Thus, our findings should be interpreted as associative rather than causal, and they support efforts to avoid unnecessary exposure to MV whenever clinically feasible and safe.

Within this line, our data may serve for benchmarking purposes for middle‐income regions and other international neonatal networks.

Further studies are required to assess the effects of invasive and different strategies of noninvasive ventilation separately.

## 5. Conclusions

In this large, multicenter cohort of VLBW infants, MV use and longer duration were associated with lower SWMM across all GAs. However, these findings should be interpreted cautiously, as MV likely reflects underlying illness severity. Strategies aimed at reducing unnecessary exposure to invasive ventilation and promoting early transition to noninvasive support when clinically feasible may improve outcomes in this population.

## Funding

No funding was received for this manuscript.

## Disclosure

A preprint version of this article has been previously published: Toso A. et al. Mechanical ventilation and survival without major morbidities in Very‐Low‐Birth‐Weight Infants: A multicenter study. Authorea. May 23, 2023 (doi:10.22541/au.168480380.00220187/v1) [31].

## Conflicts of Interest

The authors declare no conflicts of interest.

## Data Availability

The data that support the findings of this study are available from the corresponding author upon reasonable request.
